# The Effects of Adverse Childhood Experiences and Warfare Exposure on Military Sexual Trauma Among Veterans

**DOI:** 10.1177/08862605221109494

**Published:** 2022-08-12

**Authors:** Carly E. Doucette, Nicole R. Morgan, Keith R. Aronson, Julia A. Bleser, Kimberly J. McCarthy, Daniel F. Perkins

**Affiliations:** 1 Pennsylvania State University, University Park, PA, USA

**Keywords:** military sexual trauma, adverse childhood experiences, military warfare or combat, sexual attention or contact, veterans

## Abstract

Military sexual trauma (MST) is a pervasive problem; this study examined the relationship of the precursory traumas of adverse childhood experiences (ACEs) and warfare exposure with MST. Post-9/11 veterans were surveyed at 3 months and at 24 to 30 months post-military separation. Female veterans who experienced at least 1 ACE but no warfare exposure were significantly more likely to receive unwanted sexual attention. Veterans (males and females) experiencing three or more ACEs but no warfare exposure were significantly more likely to receive unwanted sexual attention and contact. Experiencing only warfare exposure was not related to unwanted sexual attention or contact for females; however, a significant interaction was found between combined warfare exposure, ACEs, and MST for males and females. Veterans who reported warfare exposure and one to two or three or more ACEs were more likely to report unwanted sexual attention and/or contact. Exploration of individual ACEs revealed a significant relationship between childhood sexual abuse and unwanted sexual contact. For females, witnessing domestic violence predicted unwanted sexual contact. There was also a significant interaction between childhood sexual abuse and warfare exposure. Females who experienced both childhood sexual abuse and warfare exposure were significantly more likely to receive unwanted sexual attention and unwanted sexual contact. Albeit a small sample, males who experienced both were also significantly more likely to receive unwanted sexual attention. The findings reveal that precursory traumatic experiences in childhood and the interaction of ACEs and warfare exposure during military service can increase the likelihood of unwanted sexual attention and contact. This research further substantiates the need for screening efforts. It also demonstrates the importance of practitioners engaging in trauma-informed care principles and practices to address the residual effects of previous experiences during sexual trauma or mental health treatment efforts.

## Background

The cumulative risk model for military sexual trauma (MST) can be further delineated to tailor interventions and improve the well-being of military veterans. MST is defined by federal law as “psychological trauma. . .resulted from a physical assault of sexual nature, batter of a sexual nature, or sexual harassment which occurred while the Veteran was serving on active duty, active duty training, or inactive duty training” (Title 38 U. S. Code 1720D). In recent decades, high-profile MST events prompted congressional mandates for the creation of a reporting hotline and sexual trauma treatment teams to address MST experienced by military service members and reduce the well-documented, deleterious effects of MST on their health and well-being (Burgess et al., 2013). Post-9/11 deployed veterans who screen positive for MST have been found to be three times more likely to have a mental health condition than those who screen negative (Kimerling et al., 2010). Compared to females with no MST victimization, those experiencing MST were four times more likely to develop post-traumatic stress disorder (PTSD) and three times more likely to develop depressive disorders (Kimerling et al., 2010). Similarly, males experiencing MST were approximately two and half times more likely to develop PTSD and depressive disorders compared to males with no MST history (Kimerling et al., 2010). Furthermore, MST increases the risk of suicide attempts and deaths (Kimerling et al., 2016).

Department of Defense (DoD) reports indicate increases in rates of reported sexual assault over the past 10 years (Department of Defense [DoD], 2019). However, approximately two-thirds of cases are estimated to go unreported (DoD, 2018) for a variety of reasons (i.e., fear of retaliation). MST prevalence varies across studies (Hyun et al., 2009; Wilson, 2018). A meta-analysis found that approximately 15.7% to 31.2% of military service members and veterans reported MST (23.6%−52.5% of women and 1.9%−8.9% of men); percentages varied depending on the definitional parameters of sexual harassment and assault (Wilson, 2018). Although proportionally more females are victims of MST, some studies have found that the absolute number of males and females who report MST are similar, which may suggest males are an under-emphasized but substantial at-risk population; a need exists to further explore rates of male victimization (Hyun et al., 2009; Kimerling et al., 2007, 2010; O’Brien et al., 2015).

Due to the negative effects and prevalence of MST, the Veterans Health Administration (VHA) has adopted universal MST screening procedures and other responses (e.g., hotlines, treatment teams; Kimerling et al., 2008). DoD and VHA initiatives are helping to address MST; however, collecting more information will empower researchers in identifying risk factors associated with MST victimization. Although harassment and assault are never the victims’ fault or responsibility, understanding who is at greatest risk for being targeted, and subsequently harmed, will help to better inform prevention and intervention efforts. The aim of this study was to examine risk factors of MST victimization within a large, recently separated sample of post-9/11 veterans.

### Accumulation of Risk Factors and Subsequent MST

Early-traumatic life experiences, coupled with adult risk factors, may interact, or compound to increase the risk of experiencing MST. Causal pathways to adult sexual victimization are complicated. Cumulative risk models suggest that individuals who experience increased levels of adversity, overall, are more likely to have negative outcomes (Rutter, 1979). Furthermore, adversity may have a threshold effect; when an individual reaches a sufficient “dose” or has met a threshold, he or she, then, experiences more negative outcomes (Felitti et al., 1998; Giovanelli et al., 2016; Perkins & Borden, 2003; Rutter, 1979). Most research has been conducted on civilian populations with an emphasis on environmental factors such as poverty, social support, stress, and adult adversity (Jones et al., 2018). Numerous studies have examined the effects of household dysfunction and child maltreatment on adult outcomes (Felitti et al., 1998; Giovanelli et al., 2016; Horan & Widom, 2015; Perkins & Jones, 2004). Childhood experiences may propel individuals into at-risk or high-stress adult environments (Giovanelli et al., 2016; Jones et al., 2018). The military context creates considerable stress and risk for military service members, especially those involved in combat-related positions such as combat patrols and witnessing the collateral damage of war (Aronson et al., 2020).

#### Adverse childhood experiences

One prominent risk factor for victimization is past victimization, including exposure to adverse childhood experiences (ACEs) (Desai et al., 2002; Gabor & Mata, 2004). ACEs are life adversities that occur before the age of 18 and include experiencing child neglect or maltreatment (i.e., emotional/psychological, physical, or sexual abuse); witnessing violence within the household or community; living with family members who struggle with mental health issues or substance misuse; parental separation or incarceration; and exposure to environments that lack safety, stability, or bonding opportunities (Felitti et al., 1998; Merrick et al., 2017). Researchers have increased their focus on ACEs exposure among service members and veterans amidst concerns that the prevalence of ACEs may be higher in military populations (Katon et al., 2015). In two population-based studies, Blosnich et al. (2014) found that 27.3% of volunteer-era veterans reported four or more ACEs, and Aronson et al. (2020) found that 12.3% of male veterans and 24.5% of female veterans also reported four or more ACEs. These prevalence rates, when compared to the approximately 15% of middle-class civilians who experience four or more ACEs (Felitti & Anda, 2010), demonstrate that those in the military may be more likely to experience ACEs. Furthermore, having a non-supportive family and experiencing child maltreatment were significant predictors of MST for male veterans of Gulf War I (Murdoch et al., 2014; Suris & Lind, 2008). Individuals may enter the military to escape from negative circumstances or dysfunctional childhood homes (Blosnich et al., 2014). These individuals are typically younger and in the enlisted paygrades, both of which are risk factors for MST (Kimerling et al., 2011; Klingensmith et al., 2014; Murdoch et al., 2014; Scott et al., 2014).

#### Warfare experiences

In addition, specific military events and settings contribute to increased risk of MST (Barth et al., 2016; Burns et al., 2014; Murdoch et al., 2014; Sadler et al., 2003). These risk factors include being on active duty status, being enlisted in the Navy or Marines (Barth et al., 2016; DoD, 2018; Klingensmith et al., 2014), living in crowded conditions, and the unit’s tolerance of sexual harassment (Murdoch et al., 2014; Sadler et al., 2003). Complicating matters further, members of the military also experience unique traumatic stressors related to warfare (Wang et al., 2015), which may include exposure to combat, witnessing the aftermath of battle, perceiving threats, and living and working in difficult environments (King et al., 2006). Exposure to these stressors increases the risk of MST (Barth et al., 2016; Murdoch et al., 2014). With the recent expansion of combat occupations to female service members and the changing types of warfare exposure across different eras, examination of a sample of recent veterans was needed to better understand how present-day combat experiences predict MST.

#### Other risk factors

Within the empirical literature, other personal factors relate to the risk of being sexually victimized in the military. Race and ethnicity play a role in MST victimization, and those who are non-White are at a higher risk for victimization (Klingensmith et al., 2014; Scott, 2014). Marital status is also related to MST; those who are victims of MST are less likely to be married (DoD, 2019; Kimerling et al., 2007). Findings are complicated when examining gender, especially for males. In the DoD’s most recent annual report on MST, females reported their attackers were nearly exclusively male and acted alone (92%), whereas males reported being victimized by men, women, or both (DoD, 2019). Males were three times as likely to view their incidents as part of a hazing ritual or bullying than their female counterparts (DoD, 2019). These differences demonstrate a qualitatively different experience of MST; therefore, the predictors of MST may be distinct by gender.

Military paygrade should also be considered, as it may contribute to hierarchy and power dynamics; it also serves as a proxy for age, time in service, and education. Paygrades include “enlisted,” “warrant officer,” and “officer” and are denoted by an “E,” “W,” “O,” and a numeric value that represents the achievement of testing for promotion (DoD, 2022). The lowest paygrade is “E-1,” whereas the highest paygrade is an officer labeled as “O-10.” Enlisted service members require a minimum of a high school diploma. A warrant officer is considered a highly trained specialist with several years of experience as an enlisted service member. A commissioned officer requires a bachelor’s degree or higher.

## Current Study

Based on the prevalence data, sexual victimization and harassment are not rarities within the military, and MST’s well-established deleterious effects on the health and well-being of service members demand more attention be given to the predictors of MST (DoD, 2018; Kelly et al., 2008; Suris & Lind, 2008). Understanding the nuanced relationship between precursory traumatic or high-stress experiences (i.e., ACEs or warfare exposure) and MST is essential, as the threshold effect of risk factors for negative outcomes has been consistently demonstrated within the ACEs literature (e.g., Felitti et al., 1998; Giovanelli et al., 2016; Horan & Widom, 2015) including studies that examine military populations (e.g., Aronson et al., 2020; Scoglio et al., 2019).

The current study of a large sample of recently transitioning post-9/11 veterans undertook an in-depth examination of potential risk factors for experiencing MST separately for male and female veterans. The study sought to expand upon the current literature by examining the association of ACEs, warfare exposure, and the interaction of ACEs and warfare exposure on the occurrence of MST. This examination provided a deeper exploration into the cumulative risk model of MST. Finally, this study also explored the individual direct effect of specific ACEs on MST.

Drawing from prior research, the study researchers hypothesized that (1) veterans who experienced ACEs or warfare exposure would be more likely to experience MST than those who did not experience trauma (ACEs or warfare exposure). Moreover, (2) veterans who had experienced both warfare exposure and ACEs would be more likely to experience MST than those who did not experience trauma. Finally, given prior research that demonstrates child maltreatment, particularly child sexual abuse, is a risk factor for adult victimization (Ports et al., 2016), it was hypothesized that (3) individual’s reports of ACEs that reflected specific child maltreatment (sexual abuse) would significantly predict MST.

## Methods

### Procedures

#### Recruitment

Details on the recruitment procedures used in this study have been previously published (Vogt et al., 2018). In brief, each participating veteran was identified from the Veterans Affairs/Department of Defense Identity Repository and had separated from the military in 2016. Initial surveys were completed within 3 months after discharge or deactivation from active status. Incentives were provided for survey completion. The online survey took approximately 45 minutes to complete. Five additional waves of surveys were administered at 6-month intervals. The Institutional Review Board at ICF International, Inc. approved the study, and informed consent was provided by participants.

#### Participants

Of the total invited population of 48,965 veterans, complete data were provided by 9,566 veterans. Veterans who completed wave 5 of the survey, when ACEs were assessed, were included in the current analysis (*n* = 5,792). Weighted analysis was conducted to generalize to the invited population (weighted *n* = 48,484). The assessment in wave 5 allowed the analysis to correct for differential attrition that may have occurred since the initial assessment (Chen et al., 2015). Participants were mostly male (84%) and non-Hispanic White (63%). Participants had served as members of the Army (32%), Navy (19%), Air Force (14%), Marine Corps (17%), and the National Guard/Reserves (18%). Paygrade of participants included 41% E1 to E4, 30% E5 to E6, 13% E7 to E9, 1% W1 to W5, 6% O1 to O3, and 8% O4 to O10. Among participants who completed wave 5 of the survey, their average age at wave 1 was 32.2 years. Tables 1 and 2 provide more demographic details, and the reported incidences of ACEs and warfare exposure. A further detailed reporting of sample characteristics is available; see Vogt et al. (2018) and Aronson et al. (2020).

**Table 1.** Descriptive Statistics: Incidence of ACEs, Warfare Exposures, and Mental Health Outcomes in the Sample.

	Females *n* = 7,741		Males *n* = 40,743	
	Estimate (%)	Design effect	Estimate(%)	Design effect
Unwanted attention	40.8	1.20	3.2	1.20
Unwanted contact	17.5	1.20	1.1	1.19
Adverse childhood experiences				
Emotional abuse	39.4	1.20	25.1	1.17
Family history of mental illness or alcohol abuse	36.5	1.18	22.1	1.18
Emotional neglect	32.2	1.20	18.1	1.18
Physical abuse	27.2	1.21	16.8	1.16
Sexual abuse	23.2	1.24	5.6	1.13
Domestic violence	16.8	1.24	10.2	1.13
Physical neglect	15.5	1.25	10.0	1.22
No ACEs, no warfare (reference group)	29.5	1.25	31.4	1.26
1–2 ACEs, no warfare	18.4	1.16	9.9	1.34
3 or more ACEs, no warfare	22.7	1.24	7.3	1.31
No ACEs, warfare	11.9	1.10	29.3	1.13
1–2 ACEs, warfare	7.1	1.00	11.7	1.07
3 or more ACEs, warfare	10.5	1.16	10.4	1.05

*Note.* Warfare = combat patrols and corollaries of combat. Analytic weighted sample with ACEs presented.

**Table 2.** Odds Ratios for Unwanted Sexual Attention/Unwanted Sexual Contact for Female Veterans—ACEs and Combat Interaction (Weighted).

	Females—unwanted sexual attention				Females—unwanted sexual contact			
	OR	95% CI		*p*	OR	95% CI		*p*
		*LL*	*UL*			*LL*	*UL*	
Paygrade E1–E4 (reference group)								
Paygrade E5–E6	1.10	0.76	1.57	.62	0.99	0.63	1.55	.96
Paygrade E7–E9	1.85	1.09	3.14	.02^a^	1.61	0.85	3.05	.14
Paygrade W1–W5	5.56	0.92	33.64	.06	2.50	0.51	12.30	.26
Paygrade O1–O3	1.01	0.60	1.71	.97	0.90	0.43	1.87	.77
Paygrade O4–O7	0.81	0.47	1.41	.46	0.69	0.33	1.42	.31
Honorable discharge (reference group)								
General/Other than honorable discharge	1.15	0.45	2.91	.77	3.72	1.44	9.61	.01
Medical discharge	0.76	0.45	1.28	.30	0.88	0.46	1.68	.70
Service support occupation (reference group)								
Combat occupation	0.99	0.46	2.12	.98	1.51	0.67	3.42	.32
Combat support occupation	1.17	0.86	1.59	.31	1.30	0.89	1.90	.17
White, non-Hispanic (NH) (reference group)								
Black, NH	0.49	0.32	0.75	<.001	0.58	0.33	1.04	.07
Hispanic	0.88	0.58	1.34	.55	0.78	0.46	1.32	.35
Asian Hawaiian Pacific Islander, NH	0.42	0.21	0.83	.01	0.57	0.24	1.35	.20
More than 1 race, NH	1.01	0.55	1.83	.99	1.55	0.82	2.91	.17
Other race, NH	0.63	0.20	2.00	.44	1.07	0.33	3.54	.91
Marital status—married first time (reference group)								
Marital status—single never married	1.75	1.03	2.96	.04^a^	1.10	0.57	2.13	.77
Married second time or more	1.28	0.84	1.96	.25	1.58	0.95	2.63	.08
Separated, widowed, or divorced	1.66	1.00	2.76	.05	1.00	0.52	1.91	1.00
Married to a veteran	1.35	0.81	2.23	.25	1.28	0.69	2.39	.44
No ACEs, no warfare (reference group)								
1–2 ACEs, no warfare	2.23	1.45	3.42	<.001	1.44	0.79	2.61	.23
3 or more ACEs, no warfare	2.65	1.76	3.99	<.001	2.04	1.20	3.46	.01
No ACEs, warfare	1.31	0.77	2.22	.31	0.91	0.44	1.85	.79
1–2 ACEs, warfare	3.04	1.73	5.32	<.001	1.93	0.97	3.83	.06
3 or more ACEs, warfare	5.14	2.99	8.83	<.001	3.32	1.71	6.44	<.001

*Note*. Weighted estimates presented: females (*N* = 7,741).

*OR* = odds ratio; CI = confidence interval; LL = lower limit; UL = upper limit. Non-substantive covariates (e.g., branch, married to a service member currently serving) were excluded from the table.

a The Bonferroni corrected *p*-value = 0.013. Any significant values above .013 should be interpreted with caution.

### Measures

#### Adverse childhood experiences

ACEs were measured using a seven-item questionnaire utilized in prior research assessing the impact of ACEs on the development of post-deployment PTSD (LeardMann et al., 2010). Assessed ACEs included physical, emotional, and sexual abuse; physical and emotional neglect; witnessing domestic violence; and living with a family member who struggles with substance abuse or mental illness. In wave 5 of data collection (approximately 24–27 months after separation or deactivation), participants were asked to retrospectively indicate whether they had experienced any of seven ACEs prior to the age of 17 years. Items are dichotomous and were summed to create the total number of experiences. Categories of no ACEs, 1 to 2 ACEs, and 3 or more ACEs were used in the analytic model, which is consistent with prior research that indicates a threshold effect for ACEs (Felitti et al., 1998).

#### Warfare exposure

A nine-item scale asked how often the veteran encountered a variety of combat-related events. These events included experiences such as encountering unexpected explosive devices or booby traps, engaging in fire fights, and seeing severely wounded or disfigured civilians or unit members/allies. Due to non-normal distribution, response options were recoded into a dichotomized variable: 1 (*at least once*) and 0 (*never*).

#### Demographics

Demographic variables, including military service variables, were used in the analysis. These included service branch, paygrade, discharge status, military occupation, race/ethnicity, and marital status.

#### Military sexual trauma

Two questions measured MST: “While you were in the military, did you receive unwanted, threatening, or repeated sexual attention, such as touching, cornering, pressure for sexual favors, or inappropriate verbal remarks?” and “While you were in the military, did you have sexual contact against your will or when you were unable to say no (for example, after being forced or threatened or to avoid other consequences)?” These questions are consistent with the universal MST screener utilized by the VHA (Kimerling et al., 2008). The first item utilized in the present study yielded a sensitivity of 0.92 and a specificity of 0.89 in prior research (Kimerling et al., 2008). The second item had a sensitivity of 0.89 and a specificity of 0.90 (Kimerling et al., 2008).

### Data Analytic Approach

Data were weighted to adjust for population estimates based on three demographic factors (i.e., branch, paygrade, and gender) and were multiplied by the non-response weight at wave 5, which was when survey questions about ACEs were asked. Frequencies were used to describe MST, types of ACEs, and the interaction of veterans’ exposure to ACEs and types of warfare exposure (see Table 1). Logistic regression analysis was used to estimate the relationship between ACEs exposure, military-related stressors, and unwanted sexual attention/unwanted sexual contact after controlling for factors associated with mental health functioning among veterans (e.g., service-connected disability, other than honorable discharge, and socioeconomic status). Data from female and male veterans were examined separately.

## Results

### Differences in MST Frequency by Gender

#### Females

For female veterans, 40.8% of the sample reported receiving unwanted sexual attention, and 17.5% reported receiving unwanted sexual contact during their military service. Female veterans of E7 to E9 paygrades were 85% more likely to experience unwanted sexual attention compared to female veterans of lower paygrades (E1–E4) (see Table 2). Note that uncorrected *p-*values are reported. Female veterans who received a general or other than honorable discharge were 3.72 times more likely to report unwanted sexual contact than those females who received an honorable discharge. There were no relationships for females’ experiences with MST between combat occupations and combat support occupations compared to service support occupations. Female veterans who were Black non-Hispanic or Asian Hawaiian Pacific Islander non-Hispanic were less likely to report unwanted sexual attention compared to White non-Hispanic female veterans. There were no significant differences by female veterans’ race/ethnicity for unwanted sexual contact. Female veterans who were single, never married were 75% more likely to experience unwanted sexual attention compared to veterans who were married for the first time.

#### Males

For male veterans, 3.2% reported receiving unwanted sexual attention, and 1.1% reported unwanted sexual contact during their military service. Male officers (O4–O7) were less likely to report unwanted sexual attention compared to male enlisted members of paygrades E1 to E4 (see Table 3). There were no relationships for males’ experiences with MST between combat occupations and combat support occupations compared to service support occupations. Hispanic male veterans and those identifying as multiracial or “other race” were 2 times, 2.36 times, and 3.87 times, respectively, more likely to report unwanted sexual attention than White non-Hispanic male veterans. Hispanic male veterans were 2.3 times more likely to report unwanted sexual contact compared to White non-Hispanic male veterans. Male veterans who were separated or divorced were 2.63 times more likely to experience unwanted sexual contact compared to male veterans who were married for the first time.

**Table 3.** Odds Ratios for Unwanted Sexual Attention/Unwanted Sexual Contact for Male Veterans—ACEs and Combat Interaction (Weighted).

	Males—unwanted sexual attention				Males—unwanted sexual contact			
	OR	95% CI		*p*	OR	95% CI		*p*
		*LL*	*UL*			*LL*	*UL*	
Paygrade E1–E4 (reference group)								
Paygrade E5–E6	0.85	0.54	1.36	.51	0.66	0.31	1.37	.26
Paygrade E7–E9	0.72	0.40	1.31	.28	0.48	0.20	1.18	.11
Paygrade W1–W5	0.42	0.09	1.87	.25	0.73	0.09	5.85	.77
Paygrade O1–O3	0.93	0.43	2.03	.86	1.07	0.31	3.78	.91
Paygrade O4–O7	0.40	0.19	0.86	.02^a^	0.24	0.05	1.16	.08
Honorable discharge (reference group)								
General/Other than honorable discharge	0.74	0.28	1.95	.55	0.12	0.02	1.02	.05
Medical discharge	0.81	0.38	1.77	.60	0.68	0.22	2.17	.52
Service support occupation (reference group)								
Combat occupation	0.81	0.49	1.34	.41	0.70	0.29	1.69	.43
Combat support occupation	0.88	0.57	1.33	.53	0.60	0.29	1.23	.16
White, non-Hispanic (NH) (reference group)								
Black, NH	1.84	1.01	3.35	.05	1.72	0.64	4.59	.28
Hispanic	2.08	1.34	3.25	<.001	2.30	1.11	4.79	.03^a^
Asian Hawaiian Pacific Islander, NH	0.89	0.31	2.55	.82	2.29	0.73	7.25	.16
More than 1 race, NH	2.36	1.19	4.71	.01	1.34	0.34	5.26	.68
Other race, NH	3.87	1.27	11.77	.02^a^		omitted		
Marital status—married first time (reference group)								
Marital status—single never married	0.88	0.52	1.48	.62	0.63	0.25	1.62	.34
Married second time or more	0.94	0.54	1.61	.81	0.90	0.38	2.14	.81
Separated, widowed, or divorced	1.37	0.78	2.41	.27	2.63	1.16	5.96	.02^a^
Married to a veteran	0.92	0.48	1.79	.82	1.28	0.45	3.60	.64
No ACEs, no warfare (reference group)								
1–2 ACEs, no warfare	1.63	0.77	3.45	.20	0.97	0.26	3.69	.97
3 or more ACEs, no warfare	4.60	2.37	8.92	<.001	5.13	2.02	13.04	<.001
No ACEs, warfare	1.96	1.07	3.57	.03^a^	1.08	0.41	2.85	.88
1–2 ACEs, warfare	2.50	1.25	5.00	.01	0.93	0.25	3.39	.91
3 or more ACEs, warfare	3.74	1.98	7.06	<.001	2.74	1.04	7.23	.04^a^

*Note*. Weighted estimates presented: males (*N* = 40,743). *OR* = odds ratio; CI = confidence interval; LL = lower limit; UL = upper limit. Non-substantive covariates (e.g., branch, married to a service member currently serving) were excluded from the table.

a The Bonferroni corrected *p*-value = .013. Any significant values above .013 should be interpreted with caution.

### Hypothesis 1: Impact of Either Warfare or ACEs Exposure on MST

Study findings partially supported the hypothesis that those who experienced either ACEs or warfare exposure were more likely to have experienced MST. Greater numbers of ACEs were associated with MST for both male and female veterans.

Female veterans who experienced one or two ACEs without warfare exposure were 2.23 times more likely to experience unwanted sexual attention compared to female veterans not exposed to ACEs or warfare (see Table 2). For findings reported hereafter, the comparison group is female veterans who experienced no ACEs and no warfare exposure. Females’ exposure to one to two ACEs without warfare exposure was not associated with unwanted sexual contact. Females who reported three or more ACEs were 2.65 times more likely to receive unwanted sexual attention and 2.04 times more likely to receive unwanted sexual contact. Females’ exposure to warfare in the absence of ACEs was not related to unwanted sexual attention or contact (see Table 2).

Male veterans who experienced one or two ACEs without warfare exposure were not more likely to experience unwanted sexual contact or unwanted sexual attention compared to male veterans not exposed to ACEs or warfare (see Table 3). For findings reported hereafter, the comparison group is male veterans who experienced no ACEs and no warfare exposure. Males reporting three or more ACEs without warfare exposure were 4.60 times more likely to receive unwanted sexual attention and 5.13 times more likely to receive unwanted sexual contact. Males with warfare exposure and no ACEs were 1.96 times more likely to receive unwanted sexual attention, but there was no relationship to unwanted sexual contact (see Table 3).

### Hypothesis 2: Impact of Both ACEs and Warfare Exposure on MST

Study results partially supported the second hypothesis that veterans who had experienced both warfare exposure and ACEs would be more likely to have experienced MST than those who did not experience trauma. These experiences differed across ACEs “dosage.”

Female veterans who reported both warfare exposure and one to two ACEs were 3.04 times more likely to report unwanted sexual attention, but there was not a relationship to unwanted sexual contact (see Table 2). Female veterans who experienced both warfare exposure and three or more ACEs were 5.14 times more likely to experience unwanted sexual attention and were 3.32 times more likely to report unwanted sexual contact compared to those who were not exposed to either warfare or ACEs.

Male veterans with warfare exposure and one to two ACEs were 2.50 times more likely to experience unwanted sexual attention, but there was not a relationship to unwanted sexual contact (see Table 3). Male veterans who experienced both warfare and three or more ACEs were 3.74 times more likely to experience unwanted sexual attention and were 2.74 times more likely to experience unwanted sexual contact.

### Hypothesis 3: Specific Child Maltreatment, Warfare Exposure, and MST

Data findings partially supported the third hypothesis that exposure to specific child maltreatment would significantly predict MST. Female veterans who reported a history of sexual abuse as a child were 88% more likely to report unwanted sexual attention compared to female veterans who did not report a history of childhood sexual abuse (see Table 4). Female veterans who witnessed domestic violence as a child were 80% more likely to experience unwanted sexual contact compared to female veterans who did not witness childhood domestic violence. Male veterans who reported a history of sexual abuse as a child were 2.04 times more likely to report unwanted sexual attention compared to male veterans who did not report childhood sexual abuse (see Table 5). For males, no specific type of ACEs had a significant main effect for predicting unwanted sexual contact.

**Table 4.** Odds Ratios for Unwanted Sexual Attention/Contact—Specific Types of ACEs for Female Veterans.

Types of ACEs	Females—unwanted sexual attention				Females—unwanted sexual contact			
First main effect model	OR	95% CI		*p*	OR	95% CI		*p*
		*LL*	*UL*			*LL*	*UL*	
Combat exposure	1.53	1.10	2.13	.01	1.31	0.87	1.99	.20
Physical neglect	0.96	0.59	1.57	.87	0.78	0.42	1.45	.44
Emotional neglect	1.28	0.84	1.95	.25	0.96	0.56	1.64	.89
Physical abuse	0.92	0.58	1.47	.73	1.04	0.63	1.73	.88
Emotional abuse	1.45	0.95	2.20	.08	1.72	0.96	3.09	.07
Sexual abuse	1.88	1.30	2.75	<.001	1.23	0.79	1.90	.36
Domestic violence	1.47	0.93	2.31	.10	1.80	1.09	2.98	.02^a^
Family history of mental illness or alcohol abuse	1.05	0.76	1.45	.76	1.11	0.74	1.69	.61
Second model (reference group: no trauma)								
No childhood sexual abuse, warfare	1.37	0.95	1.96	.09	1.08	0.68	1.71	.76
Childhood sexual abuse, no warfare	2.11	1.43	3.12	<.001	1.32	0.81	2.15	.27
Childhood sexual abuse, warfare	5.94	3.11	11.37	<.001	3.21	1.62	6.35	<.001

*Note.* Analysis conducted with weighted estimates: females (*N* = 7,741); *OR* = odds ratio; CI = confidence interval; LL = lower limit; UL = upper limit. Non-substantive covariates (e.g., branch, paygrade, discharge status, combat patrols occupation, marital status, race/ethnicity, and resilience) were excluded from the table.

a The Bonferroni corrected *p*-value = .013. Any significant values above .013 should be interpreted with caution.

**Table 5.** Odds Ratios for Unwanted Sexual Attention/Contact—Specific Types of ACEs for Male Veterans.

	Males—unwanted sexual attention				Males—unwanted sexual contact			
Type of ACEs	OR	95% CI		*p*	OR	95% CI		*p*
First main effect model^a^		*LL*	*UL*			*LL*	*UL*	
Combat exposure	1.40	0.91	2.13	.12	0.77	0.41	1.44	.41
Physical neglect	1.25	0.70	2.26	.45	1.44	0.61	3.41	.40
Emotional neglect	1.27	0.71	2.26	.42	1.28	0.59	2.78	.53
Physical abuse	1.13	0.64	2.01	.67	1.60	0.64	3.99	.32
Emotional abuse	1.47	0.86	2.50	.16	1.65	0.79	3.43	.18
Sexual abuse	2.04	1.14	3.66	.02^a^	1.54	0.61	3.88	.36
Domestic violence	0.69	0.37	1.28	.24	0.77	0.29	2.04	.59
Family history of mental illness or alcohol abuse	1.21	0.77	1.91	.41	1.17	0.54	2.52	.70
Second model (reference group: no trauma)^b^								
No childhood sexual abuse, warfare	1.61	1.04	2.50	.03^a^	1.05	0.54	2.05	.88
Childhood sexual abuse, no warfare	4.10	1.98	8.51	<.001	5.53	2.09	14.64	<.001
Childhood sexual abuse, warfare	3.43	1.67	7.06	<.001	1.09	0.25	4.81	.91

*Note.* Analysis conducted with weighted estimates: males ^a^(*N* = 40,743) ^b^(*N* = 40,775). *OR* = odds ratio; CI = confidence interval; LL = lower limit; UL = upper limit. Non-substantive covariates (e.g., branch, paygrade, discharge status, combat patrols occupation, marital status, race/ethnicity, and resilience) were excluded from the table.

a The Bonferroni corrected *p-*value = .013. Any significant values above .013 should be interpreted with caution.

Further analyses examined the interaction between childhood sexual abuse and warfare exposure for females. Warfare exposure alone (without the presence of childhood sexual abuse) was not significantly related to MST for females. A significant interaction between childhood sexual abuse and warfare exposure was found for females. Direct effects of childhood sexual abuse without warfare exposure indicated that female veterans were 2.11 times more likely to receive unwanted sexual attention compared to females with no childhood sexual abuse or warfare exposure. This finding was not related to unwanted sexual contact. Females who experienced both childhood sexual abuse and warfare exposure were 5.94 times more likely to receive unwanted sexual attention and 3.21 times more likely to receive unwanted sexual contact than females with no childhood sexual abuse or warfare exposure.

Further analyses also examined the interaction between childhood sexual abuse and warfare exposure for males. Male veterans exposed to warfare, but not childhood sexual abuse, were 61% more likely to receive unwanted sexual attention than males without childhood sexual abuse or warfare exposure. This finding was not related to unwanted sexual contact. Male veterans exposed to childhood sexual abuse but not warfare exposure were 4.10 times more likely to receive unwanted sexual attention and 5.53 times more likely to receive unwanted sexual contact than males without childhood sexual abuse or warfare exposure. Males who experienced both childhood sexual abuse and warfare exposure were 3.43 times more likely to receive unwanted sexual attention than males without childhood sexual abuse or warfare exposure. The interaction effect was not significantly predictive of male unwanted sexual contact.

## Discussion

Presently, this study is one of the few that examines the association of exposure to ACEs and combat on the occurrence of MST among both female and male veterans. The study hypothesized that either ACEs or warfare would be predictive of MST, and the hypothesis was partially supported, particularly for individuals who experienced three or more ACEs. The findings align with prior research, which has found that exposure to ACEs increases the likelihood of adult revictimization; a graded-dose response exists as those who experience more ACEs are at increased risk for adult sexual victimization (Klest, 2012; Parks et al., 2011; Ports et al., 2016). Study results suggested that the graded-dose response and cumulative effect also occurred for the veterans, and this lent support for the proposed model of cumulative risk for MST. Given ACEs cumulative effects on adult victimization (Ports et al., 2016) and their interrelated nature (Dong et al., 2004), any efforts to prevent or treat MST should include addressing ACEs as part of trauma-informed care. Of note, without ACEs exposure, warfare exposure did not significantly predict MST for female veterans. However, within this analysis, there were large standard errors, which could be the result of a third, untested, moderating variable (e.g., unit cohesion, non-warfare contexts, and gender ratios). Given that combat and military experiences are varied, considering how that variability may have impacted the present analyses is an important next step for future research. Notably, this examination does not imply that there is a direct, causal relation of ACEs on MST.

This study also hypothesized that there would be a significant interaction among exposure to ACEs and warfare combined, so veterans who were exposed to both would be more likely to experience MST. There was a significant interaction between warfare exposure and the presence of three or more ACEs and MST. Female and male veterans exposed to both warfare and three or more ACEs had an increased likelihood for MST (i.e., both unwanted sexual attention and contact).

There may be a qualitative difference in MST experiences for males and females. For male veterans, sexual harassment and assault may be motivated by the fraternal nature of the military (i.e., male power hierarchies and rigid masculine ideologies), as many male victims report their experiences were the result of hazing rituals or bullying (DoD, 2018; Wadham, 2017). Hierarchies and social approval lead to “in” and “out” group dynamics, and individuals in “out-groups” may be more likely to be targets or victims of hazing and bullying that may include sexual victimization through harassment or physical contact. “Out-groups” may include individuals who are ethnically and/or racially diverse, non-heterosexual, and non-cisgendered (Wadham, 2017). This study did find being a male veteran of some races/ethnicities increased the likelihood of experiencing MST; however, it did not specifically examine predictors such as sexuality and gender identity. In addition, given that military roles, especially combat roles, are still filled mostly by males (DoD, 2017), females in the service represent a large “out-group” that is often outnumbered and outranked. Although most females in the military do not view their MST experiences as a product of hazing or bullying (DoD, 2018), violence against females may be more normalized within the military, fraternal culture given females’ “out-group” status (Wadham, 2017). Further research can explore sexual violence as a response to gender role violations, especially among women in combat.

Wadham (2017) suggested that sexual aggression happens within the military context, in part, due to social approval and masculine hierarchy. These behaviors may be less socially acceptable or advantageous in the context of warfare exposure when one’s survival is dependent on others. Thus, de-escalation of sexually aggressive behaviors may occur during military combat missions. However, sexually aggressive individuals are unlikely to cease these behaviors totally; when the likelihood of assault decreases, perhaps, these behaviors are replaced by less-severe harassment behaviors. This study elucidated warfare exposure, even in the absence of ACEs, to be a risk factor for MST for males. The combination of warfare with ACEs increased risk of MST for both genders. Further research can disentangle deployment dynamics (e.g., socialization during rest and recovery after warfare) and military occupations that may increase risk during combat (e.g., unit isolation; few peers or bystanders to witness MST). Previous research has cited the following deployment factors as contributing to MST: duration, deprivation of sexual activity, high stress, risky behaviors (i.e., alcohol use), and wartime perceptions that alter “normal” behaviors (Burns et al., 2014).

Finally, this study hypothesized that ACEs reflecting child maltreatment would predict MST victimization, as these are known risk factors for adult sexual victimization (Ports et al., 2016). Consistent with threshold effects of cumulative risk models (Rutter, 1979), exploratory analysis of individual ACEs found limited support that one specific ACE was predictive of MST. There was a main effect of childhood sexual abuse on unwanted attention for both genders. Moreover, childhood sexual abuse produced an interaction with warfare exposure to predict MST, specifically unwanted sexual attention, across genders. For male veterans, the interaction of sexual abuse and warfare exposure was not significant for unwanted sexual contact. A small percentage of males endorsed unwanted sexual contact, which may reflect underrepresentation. Confidence intervals suggest there may be a third unmeasured moderator that can elucidate the relationship between ACEs, warfare exposure, and MST.

Prior research on revictimization has suggested that individuals who experience sexual abuse early in life are at a higher risk for sexual revictimization as adults (Ports et al., 2016). Although responsibility always lies with the perpetrator of crimes and not the victims, it is important to understand why victims are targeted or more susceptible to acts of violence to inform prevention/intervention efforts. There are several theoretical rationales for revictimization; however, research on these theories remains mixed (Breitenbecher, 2001). One theory with evidentiary support proposes that victims of child sexual abuse develop maladaptive perceptions of threat due to normalization of sexual threat through repeated exposure (Breitenbecher, 2001). These victims may develop latency in recognition of or attentional bias to sexual threat; thus, their ability to perceive and discern sexual threat is affected (Latack et al., 2017). Additionally, these individuals may experience lower self-efficacy and state dissociation, which limits their capacity to leave dangerous situations, and these characteristics have also been shown to predict revictimization (Bockers et al., 2014). Understanding these processes is very important for both veterans and the general population when considering programmatic development and decision-making for prevention and intervention.

Given the demonstrably harmful effects of MST, exploration of barriers to reporting is also essential to advancing evidence-based prevention and intervention efforts. The reasons individuals do not report MST appear to be ubiquitous and systemic including the potential for negative career impacts, retaliation, and further traumatization. Military perpetrators are sometimes higher in the chain of command and can affect the future careers of their victims (Street et al., 2008). Although policies and protections are improving, the chain of command may also conduct internal investigations where MST goes unpunished. There is also sometimes a lack of confidentiality when reporting, even when victims choose to remain anonymous (Burns et al., 2014). Those who report MST can also be met with rejection from their unit (Burns et al., 2014). In a DoD annual survey on sexual assault, 64% of females who indicated being sexually assaulted reported that they experienced at least one negative behavior because of their report, and 21% of the female victims indicated that the retaliation they experienced was expressly prohibited by military law (DoD, 2018). Thus, victims of MST are in vulnerable situations and may forgo meaningful professional help and services. Considering the deterrents to MST reporting may lead to the identification of gaps in prevention and to the provision of more effective services for victims.

There is evidence that ACEs, warfare exposure, and MST have cumulative effects on health and well-being, such as on PTSD (Scoglio et al., 2019); this demands that practitioners working with individuals who experience MST be aware of precursory trauma to inform treatment sessions’ content and goals. Evidence-based treatments, such as Prolonged Exposure and Cognitive Processing Therapy, can potentially address the intersectionality of ACEs and MST. However, as important as channels for reporting and treatment are to victims’ well-being, they are reactive strategies. The development and evaluation of MST prevention efforts within the military context is needed (Orchowski et al., 2018).

### Limitations

There are several study limitations that create an opportunity for future research. First, this study relied on retrospective reporting of both ACEs and MST. For individuals with longer military service (i.e., military retirees or officers), these experiences may have occurred decades prior to when they reported them in the present study. Second, although not atypical in the literature, the MST variables are represented by single items. Due to the cross-sectional nature of the study, there was no way to establish temporal precedent between combat experiences and MST. There were also definitional limitations regarding MST in that the measure of unwanted sexual contact did not discriminate between coercive sexual contact and forcible sexual contact. The measure also did not differentiate whether the perpetrator was a civilian or military member. Third, the proportion of the male veteran sample who reported sexual assault was low in comparison to female veterans. Thus, results from the analyses of male veterans should be interpreted with some caution, even though an unweighted sample of *n* = 90 is above the general guidance for analyses. Fourth, there were several analyses included, and this increases risk of a Type I Error related to conducting multiple statistical tests. Given the exploratory nature of this study, multiple tests of the predictors were warranted in interaction analyses. Comparison tests are not necessary for exploratory analyses (Bender & Lange, 2001). Regardless, a Bonferroni correction method was employed with the corrected *p*-value = α (the original *p-*value of .05 divided by the number of tests performed). The Bonferroni corrected *p-*value was =.013. Interpretations should be made with caution for any Bonferroni corrected *p*=value above .013. Critics of the Bonferroni correction suggest it is too conservative, and it risks a Type II Error. In addition, statistical significance is frequently assumed substantive, even though it does not inform the magnitude or meaningfulness of effect. Statistical significance and effect size complement one another, and researchers like Nuzzo (2014) suggest statisticians should not just be asking if there is an effect, but asking how much of an effect. For example, in this study, the likelihood of male veterans experiencing unwanted sexual contact was 2.7 times if male veterans experienced three or more ACEs and warfare compared to male veterans with no ACEs or warfare. Finally, this study allowed those who did not identify as cisgender to report their gender through open-ended identification; however, data from transgender and nonbinary veterans were not able to be examined due to the extremely small sample.

### Future Directions and Conclusion

Future research should examine important situational variables of combat experiences, including unit cohesion, attitudes toward sexual harassment/violence, female/male ratios of units, and specific combat occupations, to determine whether there are unique combat experiences that increase the likelihood of MST. Future studies should also examine temporal precedent regarding whether combat experiences temporally predict MST, or if the situational variables related to combat exposure predict MST. There should also be further examination into the qualitative differences of MST experiences pertaining to gender and sexuality, given the increased visibility of lesbian, gay, bisexual, transgender, and questioning (LGBTQ+) individuals in the military and their increased risk for harassment and assault. Although admittedly difficult, another future direction could investigate perpetrator behavior and characteristics, including perpetrators’ motivations, predatory behaviors such as how they chose their victims and intimidation tactics, occupations, and personal history characteristics of ACEs or victimization. This knowledge, coupled with who is at risk for being a victim of MST, can inform prevention and intervention efforts. In addition, examination of victim’s attitudes around sexually threatening behaviors and latency in risk recognition may lead to a better understanding of the causal pathways between prior victimization/ACEs and adult victimization in the military context. Furthermore, research on the effectiveness of screening and treatment protocols for MST can be explored to promote best practices for assessing and addressing prior trauma with evidence-based programs and practices.

## Appendix: Masked Version of the Narrative Description

The data reported in this article were collected as part of a larger, longitudinal data collection effort at seven points in time. Findings have been reported in separate publications. Only two of those previous journal publications explored variables related to ACEs or warfare exposure. Research questions and studied variables differed from this manuscript. The purpose of previous studies was to test an interaction between childhood traumas and warfare exposures on mental health (Morgan et al., 2022). The focus of this current article is unique in examining the relationship of MST with the traumatic experiences of ACEs and warfare exposure.
